# Brittle cornea syndrome: a case report and review of the literature

**DOI:** 10.1186/s12886-018-0903-2

**Published:** 2018-09-18

**Authors:** Qi Wan, Jing Tang, Yu Han, Qibin Xiao, Yingping Deng

**Affiliations:** 1 Department of Ophthalmology, The people’s hospital of Leshan, Leshan City, People’s Republic of China; 2 Department of Ophthalmology, West China Hospital, Sichuan University, Chengdu, Sichuan People’s Republic of China

**Keywords:** Brittle cornea syndrome, Blue sclera, Case report, Literature review, Keratoconus

## Abstract

**Background:**

To report a patient who presented with bluish scleral discoloration, keratoconus, and progressive high myopia.

**Case presentation:**

A 6-year-old Chinese female patient presented with a significant bluish discoloration of the sclera in both eyes and extreme corneal thinning with anterior corneal protrusion. General pediatric physical examination was normal for all systems and no genetic disorders known were observed.

**Conclusions:**

We aim to highlight the importance of diagnosis and treatment of patients suffering from Brittle cornea syndrome. Timely diagnosis and early provision of protective glasses seem to be the most important step in treating BCS. To our knowledge, this is the first case of BCS being reported in the Asia area.

## Background

Brittle cornea syndrome (BCS) is a rare autosomal recessive connective tissue disease that characteristically presents with corneal thinning and fragility. It’s first reported by Stein et al. [1] and propagated by Ticho et al. [2] It is generally believed that mild corneal thinning and blue sclera are associated with heterozygous mutations [3]. Because the corneal stroma is very thin, it’s very easily leads to keratoglobus, keratoconus, high myopia and irregular corneal astigmatism [4, 5]. What’s more, under the normal biomechanical stresses, corneas of BSC are unable to maintaining their shape and structural integrity and are prone to develop spontaneous rupture. Therefore, almost all the patients have lost their vision due to corneal rupture and scar [6, 7]. So, early diagnosis and treatment of BCS is very important for the patients. As far as we known, this is the first case of BCS reported in the Asia area. Unfortunately such molecular analyses were not performed in our case. However, she did not present bone fractures or deafness as in osteogenesis imperfecta. Nor did present skin or ligament hyperelastic changes as in Ehlers-Danlos syndrome, or changes in stature similar to the Marfan syndrome.

## Case presentation

A 6-year-old Chinese girl presented to People’s hospital of Leshan, Department of Ophthalmology, in August 2017. The parents reported their child with a progressive loss of vision and bluish discoloration of sclera. Family history was negative for known conspicuous eye disorders, no infections or abnormalities in pregnancy or birth, and show no genetic disorders were known. General pediatric physical examination was normal for all systems. The parents and other members of the family were all native Chinese, had no abnormality of the eyes. Overall general physical examination was normal for all systems.

**Fig. 1:**
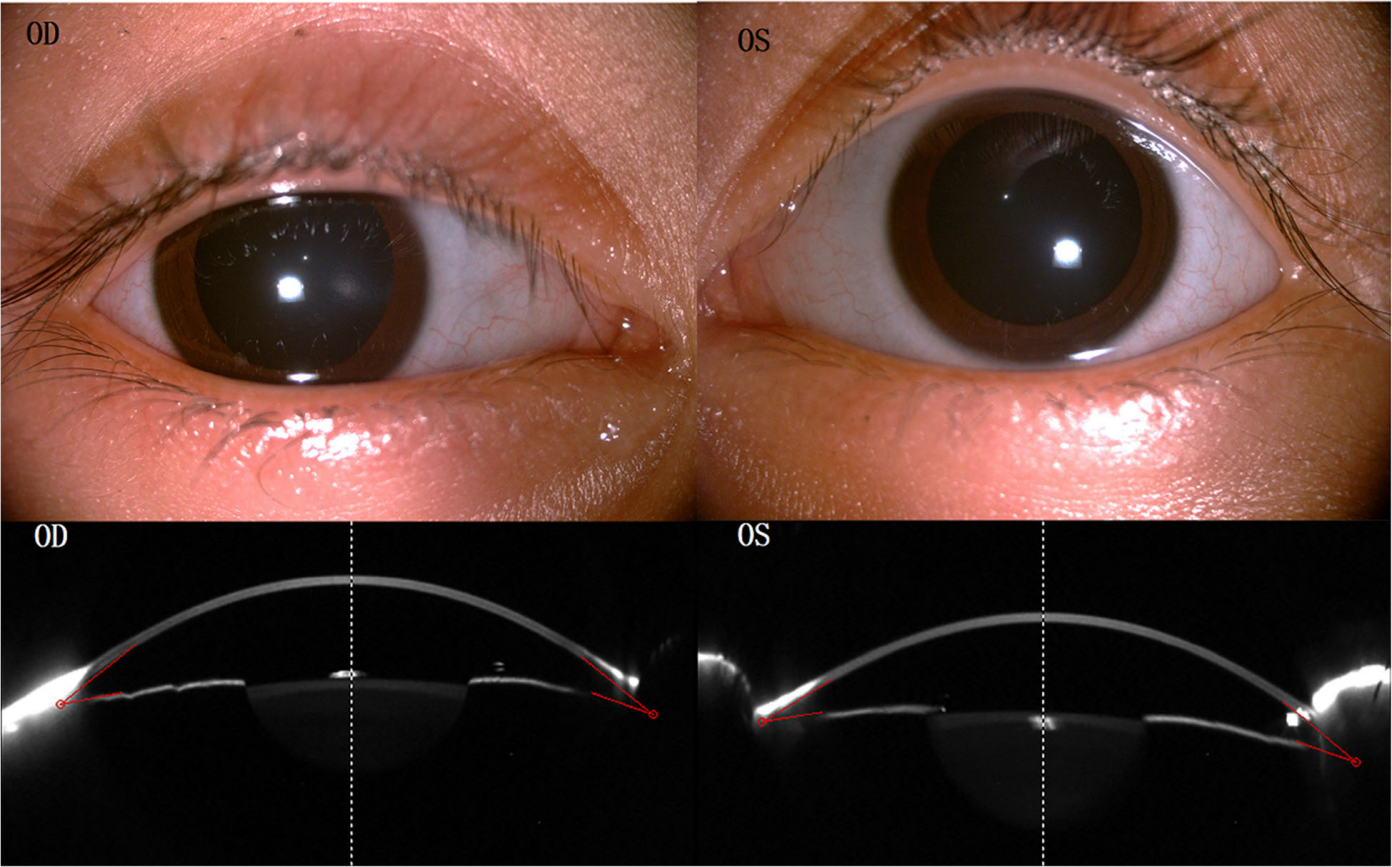
Digital photograph of the 6-year-old girl with brittle cornea syndrome,intense bluish discoloration of the sclera and keratectasia. The Scheimpflug image of cornea showed extreme corneal thinning. Central corneal thickness was 324 μm in right eye and 313 μm in the left eye

**Fig. 2:**
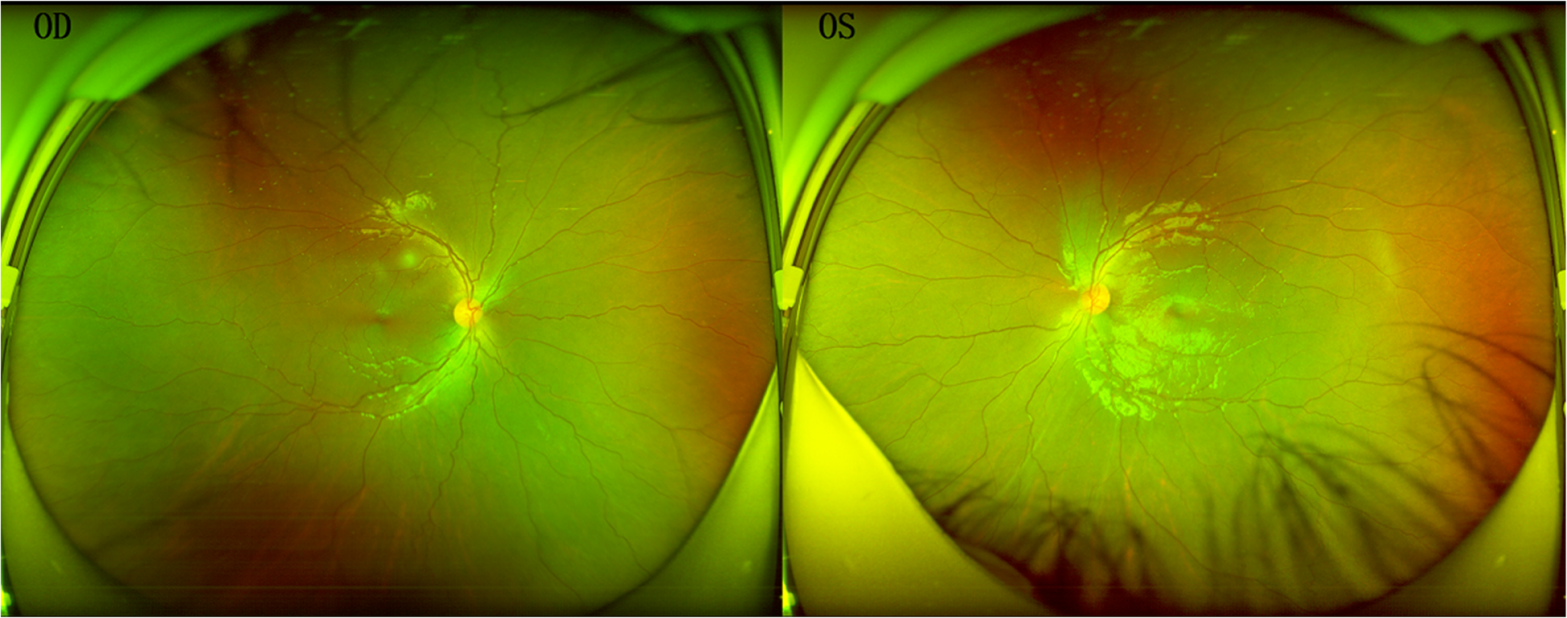
Ultra-widefield fundus imaging of case

**Fig. 3:**
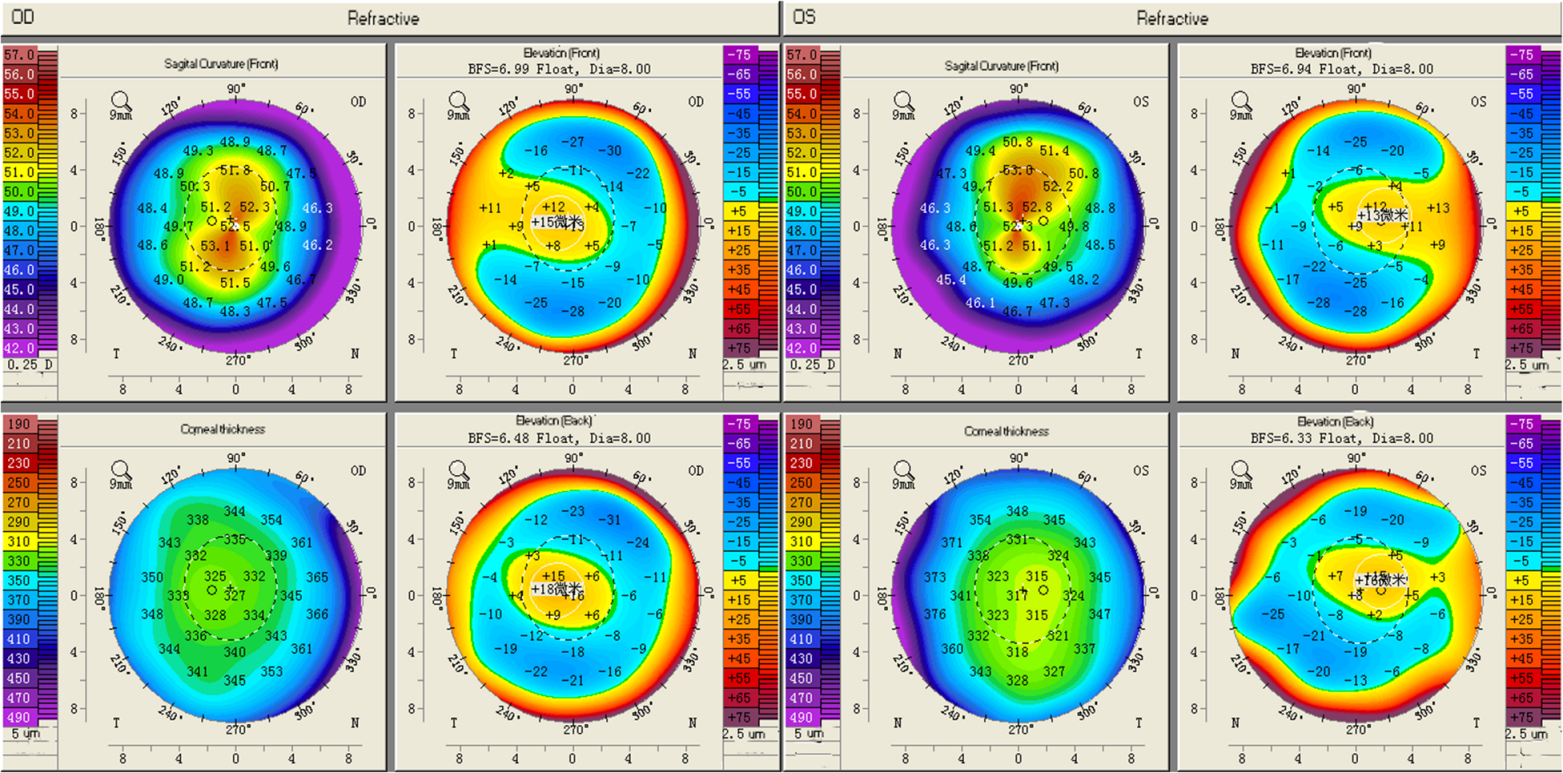
Pentacam-refractive maps of the right and left eyes of Case

On ophthalmological examination showed that the cornea were obviously prominent, with a significant bluish discoloration of the sclera in both eyes (Fig. 1). The examination of anterior segment eye manifested that an obviously thin cornea with protrusion and the posterior segment was examined by Optosmap Daytona and showed no retinal anomalies or retinal detachment (Fig. 2). Pentacam HR anterior segment tomography indicated that keratoconus with steepening in both eyes which have a maximum keratometric power of 54.10 D in the right eye and a maximum keratometric power of 54.40 D in the other eye (Fig. 3). The thinnest point of cornea assessed by Pentacam,which showed that the right eye was 324 μm thickness with corneal astigmatism in topography (− 2.6D at 163 degrees) and 313 μm thickness measured in the other eye with corneal astigmatism in topography (− 2.7D at 172 degrees) (Fig. 3).

The best corrected visual acuity of right eye was 80/200 and the manifest refraction was − 4.75 /− 2.25 × 180. In the left eye, the best corrected visual acuity was 60/200 with − 3.00/ -4.00 × 170. Intraocular pressure was within normal levels (10 mmHg in the both eyes) [8]. Axial length was 21.45 mm in the right eye and 21.33 mm in the left eye. All which suggested that she is a refractive myopia. Specular microscopy showed that the right eye with the density of 3,459 cells/mm^2^ in corneal endothelial and 3,654 cells/mm^2^ in the left eye. According to clinical symptoms and signs, the child was diagnosed with keratoconus, blue sclera, and refractive myopia. Therefore, a presumptive diagnosis of BCS can be made and referred her to the Contact Lens Department to improve her visual acuity.

## Discussion

Brittle cornea syndrome was first described by Stein et al. for two siblings of a consanguineous Tunisian Jewish family, characterized by red hair, blue sclera, and brittle cornea with recurrent spontaneous perforations [1]. By now, more than 60 patients involved white and black race have been reported [9]. However, there is no yellow race about BCS have been reported in Asia. As we know, this is the first case of BCS reported in the Asia area. Although BCS is a rare genetic disease, it’s extreme corneal thinning (220–450 μm) (normal range 520–560 μm) affected individuals are at high risk of corneal rupture, leading to irreversible blindness [1, 5, 10]. So, It is urgent for us to better understanding this disease and taking timely measures to manage and follow-up this disease.

### The genetics of BCS

Clinically, there has no clear diagnostic criteria for BCS. Therefore, early genetic detection seem to more important for the diagnosis of BCS. Previous studies showed that BCS results from mutations in one of two genes:ZNF469 (encoding zinc finger protein 469) and PRDM5 (encoding PR domaincontaining protein 5). [11–13]. Mutations in the ZNF469 gene are causative for brittle cornea syndrome type1 (BCS1) and brittle cornea syndrome type2 (BCS2) is caused by mutations in the PRDM5 gene [5, 10]. As transcriptional regulators, both ZNF469 and PRDM5 are participate in pathways regulating extracellular matrix. ZNF469 is a single exon gene located at 16q24 and produes 3953 amino acid residues. Although the functional research about ZNF469 is confined, the mutations in ZNF469 gene are thought to be associated with changes in central corneal thickness. Recent researches also confirmed that ZNF469 take an important role in anterior segment development and participate in pathogenesis of common ocular disorders such as glaucoma [14–16]. PRDM5 is located at 4q25-q26,which consisting of 16 exon genes and encoding a 630 amino acid residues [17]. Although the mechanisms by which mutations in PRDM5 cause disease are not understood, a role in extracellular matrix physiology has been suggested by the recent characterization of a PRDM5 conditional knock-out mouse [18]. What’s more, Galli GG et al. using chromatin immunoprecipitation (ChIP) – sequencing also demonstrated that a direct role for PRDM5 in the regulation of collagen genes [19]. How both genes cause BCS is not yet understood, but most published disease-causing mutations in both PRDM5 and ZNF469 are homozygous mutations.

### Diagnosis and differential diagnosis

Apart form the definitively associated with ZNF469 and PRDM5 gene mutations, there are some common clinical features in BCS patients [20]. For example, ocular features include extreme corneal thinning, blue sclera, keratoconus, keratoglobus and high myopia. Extra-ocular manifestations include deafness, joint hypermobility, skin hyperelasticity, arachnodactyly, and developmental dysplasia of the hip [5, 10]. If there is no obvious extra-ocular features, extreme corneal thinning or spontaneous ocular rupture should be highly suspected to diagnosis of BCS. Moreover, there exist some other diseases associated with brittle cornea and blue sclera like the Ehlers-Danlos syndrome, osteogenesis imperfecta, and the Marfan syndrome [2, 21, 22]. However, patients with these diseases are frequently have more pronounced generalized connective tissue manifestations. As regards this case we observed, she did not present any other systemic symptoms.

### Treatment

The mainly challenge for the treatment of BCS is relies on early diagnosis, which allows us to take prompt measures to prevent ocular rupture. Protective measures and disease education are most important, provided both protective spectacles and suitable education for patients, their parents, other caregivers and school staff about these and other lifestyle measures. Previous studies showed that patients at early diagnosis of BCS whom corneal perforation was averted by the use of special protective glasses [5, 20]. For BCS patients, any reduction of central corneal thickness should be actively recommended to wear the protective glasses, but whether it is necessary to continuous use is not clear [5]. At the early period of BCS, visual acuity often impaired by keratoglobus, keratoconus and high myopia [23, 24]. The use of contact lenses is optional, however the efficacy of correction of the irregular astigmatism caused by keratoglobus and keratoconus is limited. Furthermore, the progressive corneal thinning is frequently restricted the use of contact lenses. In cases of progressive BCS, epikeratoplasty (A partial thickness corneal graft) has been used in prevention of corneal rupture, which objective to increase the thickness of limbus to limbus and allow a penetrating keratoplasty subsequently [25, 26]. Additional corneoscleral grafting is the other option for extreme corneal thinning, which will not improve vision, but may reinforce the peripheral cornea [27]. What’s more, lots of researches have demonstrated that collagen crosslinking was an effective approach in treating progressive keratoconus in children and adults [28, 29]. However, because of the extreme thinning and frailty, the corneas in BCS patients were not suitable for a CXL procedure according to the original Dresden protocol [30]. Recently, Claude K et al. used a modified collagen crosslinking for corneal stabilization in a child with BCS have achieved encouraging preliminary results [31]. But only time will tell whether the collagen crosslinking can prevent the rupture of cornea or not.

## Conclusions

In conclusion, with this case report and review of the literature, we aim to highlight the importance in the diagnosis and treatment of patients suffered from this syndrome. Timely diagnosis and early provision of protective glasses are seem to be the most important step in treating BCS. What’s more, therapeutic methods like contact lenses, keratoplasty and collagen crosslinking are recommended, but the efficacy of treatment is unsatisfactory and limited and with serious complications.

## Abbreviation

BCS: Brittle cornea syndrome
